# Biological Mechanisms and Therapeutic Opportunities in Mammographic Density and Breast Cancer Risk

**DOI:** 10.3390/cancers13215391

**Published:** 2021-10-27

**Authors:** Maddison Archer, Pallave Dasari, Andreas Evdokiou, Wendy V. Ingman

**Affiliations:** 1 Discipline of Surgical Specialties, Adelaide Medical School, The Queen Elizabeth Hospital, University of Adelaide, Woodville 5011, Australia; maddison.archer@adelaide.edu.au (M.A.); pallave.dasari@adelaide.edu.au (P.D.); andreas.evdokiou@adelaide.edu.au (A.E.); 2 Robinson Research Institute, University of Adelaide, Adelaide 5001, Australia

**Keywords:** mammographic density, breast cancer risk, immune signaling, inflammation, hormones, fibroblasts

## Abstract

**Simple Summary:**

Primary prevention approaches are urgently needed to curb the growing global breast cancer burden. Mammographic density, also known as breast density, is one of the most significant and common independent risk factors for breast cancer. As such, it is an exciting new potential target to reduce the incidence of breast cancer; we could in the future identify women with high mammographic density at an increased risk of breast cancer and take steps to reduce that risk. However, more research is needed to understand the biological mechanisms that underlie high mammographic density and how these might be used to develop new prevention strategies. Here, we review the current knowledge around the biology of mammographic density with a particular focus on immune system factors that could provide new therapeutic targets.

**Abstract:**

Mammographic density is an important risk factor for breast cancer; women with extremely dense breasts have a four to six fold increased risk of breast cancer compared to women with mostly fatty breasts, when matched with age and body mass index. High mammographic density is characterised by high proportions of stroma, containing fibroblasts, collagen and immune cells that suggest a pro-tumour inflammatory microenvironment. However, the biological mechanisms that drive increased mammographic density and the associated increased risk of breast cancer are not yet understood. Inflammatory factors such as monocyte chemotactic protein 1, peroxidase enzymes, transforming growth factor beta, and tumour necrosis factor alpha have been implicated in breast development as well as breast cancer risk, and also influence functions of stromal fibroblasts. Here, the current knowledge and understanding of the underlying biological mechanisms that lead to high mammographic density and the associated increased risk of breast cancer are reviewed, with particular consideration to potential immune factors that may contribute to this process.

## 1. Introduction

The breast is indisputably one of the most susceptible tissues in a woman’s body to cancer. A quarter of all cancers in women originate in the breast and the global burden of this disease is increasing [1]. Primary prevention is urgently needed, and for this we need a better understanding of breast cancer risk factors. Although family history is an important risk factor for breast cancer, familial breast cancer is relatively rare. Mutations in genes encoding the tumor suppressor genes BRCA1 and BRCA2 account for approximately two percent of all breast cancers [2] and around ten percent of cases are linked to family history of the disease [3]. The majority of breast cancers are attributed to nonhereditary risk factors [4], and therefore, there is potential to reduce the burden of breast cancer through strategies that modify these. More research is required to understand the underlying biology of nonhereditary breast cancer risk factors and why the breast is so susceptible to cancer.

There are many non-hereditary risk factors for breast cancer including age, obesity, menstrual and reproductive history, and mammographic density. Mammographic density is one the strongest independent risk factors for breast cancer, accounting for 30% of breast cancer occurrences [5,6]. Women with breasts classified as ‘extremely dense’ on the breast imaging reporting and data system (BIRADS) have a four to six fold increased breast cancer risk compared to women with breasts classified as ‘mostly fatty’, when matched for age and body mass index (BMI) [6]. The underlying biological mechanisms that lead to high mammographic density and the associated increased breast cancer risk are poorly understood. However, there is some evidence that interactions between epithelium, stroma, the extracellular matrix, and immune signaling together with ovarian hormones may contribute to both elevated mammographic density and breast cancer risk [7,8,9,10].

## 2. Mammographic Density and Breast Cancer Risk

**Figure 1:**
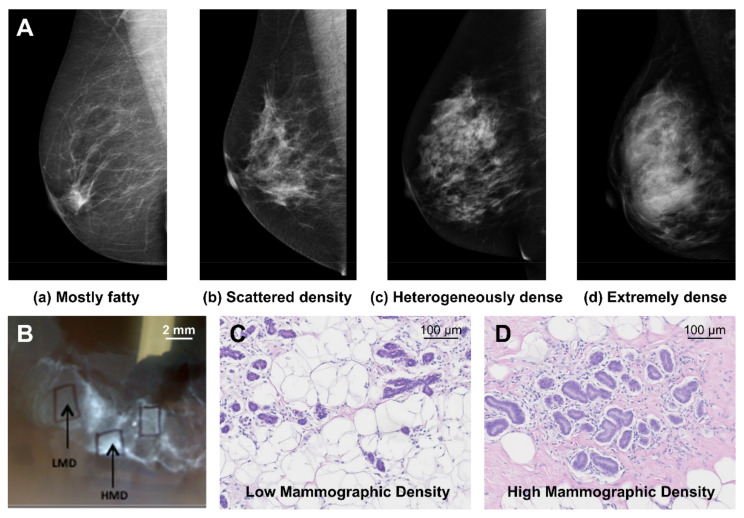
(**A**) Example images of BIRADs classification of mammographic density, (**B**) X-ray of breast tissue demonstrates areas of low and high mammographic density associated with (**C**) more adipose tissue relative to fibro glandular tissue and (**D**) more fibro glandular tissue relative to adipose tissue respectively, when hemotoxylin and eosin stained. Images reproduced with permission from [14] Elsevier and www.informd.org.au (accessed 22 October 2021).

The term ‘mammographic density’ refers to the radiographic appearance of the breast on a mammogram [11,12] and is sometimes called ‘breast density’. Mammographic density cannot be determined by self-exam and is not associated with the appearance or feel of the breast [13]. High mammographic density is indicated by a high proportion of white and bright regions on a mammogram, while low mammographic density is indicated by a high proportion of dark regions [11] (Figure 1). The white and bright regions are associated with increased abundance of fibro glandular tissue relative to adipose tissue, and conversely, the dark regions are associated with increased abundance of adipose tissue relative to fibro glandular tissue [14].

The most common method for classifying mammographic density in the clinic is the breast imaging reporting and data system (BIRADS) developed by the American College of Radiologists. This method is based on a qualitative assessment by the radiologist and classifies breasts on a scale of a to d; a is ‘mostly fatty’ where the mammogram appears mostly dark and transparent; b is ‘scattered density’; c is ‘heterogeneously dense’; and d is ‘extremely dense’ where the mammogram appears mostly white and opaque [15]. Approximately 8% of women aged between 40 and 74 have breasts classified as ‘extremely dense’, and 35% have ‘heterogeneously dense’ breasts [16], and these two categories of density are often grouped together and referred to as ‘high mammographic density’. Other quantitative and semi-quantitative methods for mammographic density classification are also used, including software programs such as Volpara and Quantra, and the Cumulus method, which is a semi-automated software program that determines a threshold to estimate the percentage mammographic density on the mammogram [17,18].

In 1976, Wolfe proposed that mammographic density is positively associated with breast cancer risk [19]. Although contentious at first, many case-control studies compounded with information on reproductive, heredity and anthropomorphic factors such as age and BMI, have supported this notion and provided evidence beyond doubt that mammographic density is one of the strongest independent risk factors for breast cancer [20,21,22]. A large meta-analysis demonstrates women with breasts classified as ‘extremely dense’ have a four to six fold increased breast cancer risk compared to women with breasts classified as ‘mostly fatty’, when matched for age and BMI [5]. Whilst this study provides the most robust evidence, it compares the two extremes of mammographic density and is not reflective of the increased breast cancer risk associated with high mammographic density above average mammographic density. More recent studies have sought to address this, and show that premenopausal women with breasts classified as ‘heterogeneously dense’ and ‘extremely dense’ have a respective 50% and 80% increased risk of breast cancer above women with breasts classified as ‘scattered density’ [6]. For postmenopausal women, the increased risk above those with breasts classified as scattered density’ is 40% and 60% for women with breasts classified as ‘heterogeneously dense’ and ‘extremely dense’ respectively.

Breast cancer is not a single disease. There are a number of different breast cancer subtypes that each have a different disease etiology and can have a different prognosis. If a risk factor elevates risk of a specific breast cancer subtype, this can provide some clues as to the underlying mechanisms that drive increased risk. Several studies have attempted to determine if there is an association between mammographic density and increased risk of a specific breast cancer subtype, however, the results of these studies have not yielded consistent results. While some studies reported that mammographic density was not specifically associated with increased risk of estrogen receptor (ER) positive breast cancer, others reported an inverse relationship with density and ER positivity and a positive relationship with progesterone receptor positive breast cancers [23,24,25,26]. However, the population analyzed in these studies were biased towards users of hormone replacement therapy, which is known to increase mammographic density [24]. Overall, the evidence suggests that high mammographic density is associated with an increased risk of all breast cancer subtypes, and the association is not greater in hormone receptor positive breast cancers or in cancers expressing HER2 [23,27].

Although it appears that there is no correlation between mammographic density and a specific subtype of breast cancer, there is some indication that mammographic density may be associated with tumour characteristics that generally indicate a poor prognosis. In a study by Yaghjyan et al., over 1000 case control pairs were examined, and reported that mammographic density measured by the Cumulus method was associated with more invasive, larger and higher-grade tumours [24]. This was consistent with another case control study by Bertrand et al. that reported an association between mammographic density and large tumour size [28]. However, several studies have demonstrated mammographic density is not associated with reduced survival in breast cancer patients [29,30,31]. One study of nearly 20,000 women reported an association between mammographic density and breast cancer mortality, after adjusting for age, BMI, menopausal status, and reproductive history [32]. However, this result might be confounded by the masking effect of high mammographic density in detection of breast cancer. High mammographic density appears white on the mammogram, and as potential tumours also appear white, high mammographic density reduces the sensitivity of mammography to detect breast cancer [33]. Therefore, the association of mammographic density with poor prognosis may be a result of high-density breast tissue masking the appearance of tumours on a mammogram, leading to later detection. High mammographic density has also been associated with a greater rate of breast cancer recurrence, and risk of recurrence is decreased when mammographic density is reduced by greater than 10%, compared to women with mammographic density that remained constant [29,34,35,36].

In summary, high mammographic density is associated with increased risk of all breast cancer subtypes. Although delayed detection of breast cancer due to the masking effect of mammographic density contributes to a poorer prognosis, high mammographic density has clearly been demonstrated to be an important independent risk factor for developing breast cancer. However, it is important to consider that the epidemiological studies that demonstrate the association between high mammographic density and breast cancer risk do not demonstrate a causal relationship. Whilst it is possible that high mammographic density causes increased risk of breast cancer, it is also possible that elevated risk of breast cancer causes high mammographic density (i.e., reverse causation). Furthermore, the association may be due to a common cause, whereby an as yet unidentified factor independently increases both mammographic density and breast cancer risk (i.e., a spurious association).

## 3. Contribution of Genetic and Environmental Factors to Mammographic Density

Many factors contribute to a woman’s mammographic density, and there are likely to be complex interactions at the biological level that ultimately determine how dense the tissue is. The relative abundance of epithelium, stroma and adipose tissue that develops in the breast during puberty largely determines the mammographic density of the adult tissue [14]. Studies on large cohorts of women that investigate relationships between mammographic density and genetic and environmental factors can provide clues to the underlying biology of mammographic density that can be further investigated in mechanistic studies. Epidemiological studies have shown that mammographic density is associated with a number of factors, including genetics, age, BMI, and reproductive history.

Mammographic density is partially inheritable. Asian and African American women are more likely to have extremely dense breasts compared to non-Hispanic white women [37,38]. Although breast cancer incidence is overall lower in China than Western countries, the rate is increasing, and high mammographic density is associated with breast cancer risk factors including increased age at first birth and shorter duration of breastfeeding [37]. Germline mutations in *BRCA1* or *BRCA2* are not associated with increased mammographic density [39] and mammographic density is considered an independent risk factor in women from high-risk families [40]. However, both mammographic density and cancer risk are correlated in sisters and in mother and daughter pairs [41]. Furthermore, studies of mammograms from monozygotic and dizygotic twin pairs reported higher correlation with monozygotic twins than dizygotic twins and the genetic contribution to mammographic density is calculated to be approximately 60% [42]. The individual genes that provide this genetic component have not been identified, and there are likely to be many that interact with environmental factors and each other to contribute to the overall mammographic density of the breast.

Age and BMI also affect mammographic density. BMI is strongly positively associated with breast area and non-dense area [20] and there is a negative association between BMI and percent mammographic density [20,43,44,45]. There is also a negative association between mammographic density and age; as age increases, mammographic density declines [46,47,48]. These inverse associations between mammographic density, BMI and age may seem paradoxical, as both increasing age and BMI are associated with increased breast cancer risk [49]. However, it is important to consider that epidemiological studies of mammographic density statistically adjust their results for the confounding factors of age and BMI to demonstrate that mammographic density is risk factor for breast cancer, independent of age and BMI.

Factors associated with a woman’s reproductive history and exposure to ovarian hormones may affect mammographic density. An inverse correlation between parity and mammographic density exists whereby there is a 4% decrease in breast cancer risk for every live birth in premenopausal women [50,51]. However, mammographic density has been reported to be higher in women where the first live birth occurred at the age of 30 or higher [51]. The association between mammographic density and breastfeeding is unclear. One study by Lope et al. reported a positive relationship between lactation and mammographic density, and greater density in women who breastfed for longer duration (longer than 9 months) [52]. Another study in Chinese women found that longer duration of lactation was associated with a lower density in postmenopausal women, but no association in premenopausal women [37].

There is evidence that mammographic density is altered by exposure to drugs that affect ovarian hormone signaling. In postmenopausal women, it is well-established that hormone replacement therapy (HRT) increases mammographic density, and also is associated with an increase in breast cancer risk [53,54,55,56,57]. Further, women who have never used HRT exhibit a greater decline in mammographic density with age than users of estrogen only and combine hormone replacement therapies [58]. Hormonal therapies for treatment of breast cancer also influence mammographic density. Treatment with tamoxifen, a drug that interferes with estrogen signaling, is associated with a decrease in mammographic density [59,60,61,62,63]. Studies on the effect of aromatase inhibitors on mammographic density have been less clear. One study reported that treatment with aromatase inhibitors reduced mammographic density and this reduction is associated with tumour free survival after at least two years of treatment [64,65]. However, some studies have found no reduction in mammographic density in response to aromatase inhibitors [66] and the use of hormonal contraceptives may not affect mammographic density [67].

It is not surprising that drugs that affect ovarian hormone signaling have a profound effect on mammographic density. Ovarian hormones provide the stimulus for breast development and as such the composition and function of the breast is fundamentally dependent on them. However, Huo et al. demonstrated that there is no difference in estrogen or progesterone receptor staining in paired tissue samples from high and low mammographic density areas within the breast [7]. Further, there is no association between high mammographic density and endogenous circulating hormones [9,44,68]. This suggests that although hormones are fundamental to mammographic density, whether or not a woman has high mammographic density is not due to the abundance or activity of endogenous hormones.

## 4. Biological Mechanisms in Mammographic Density

**Figure 2:**
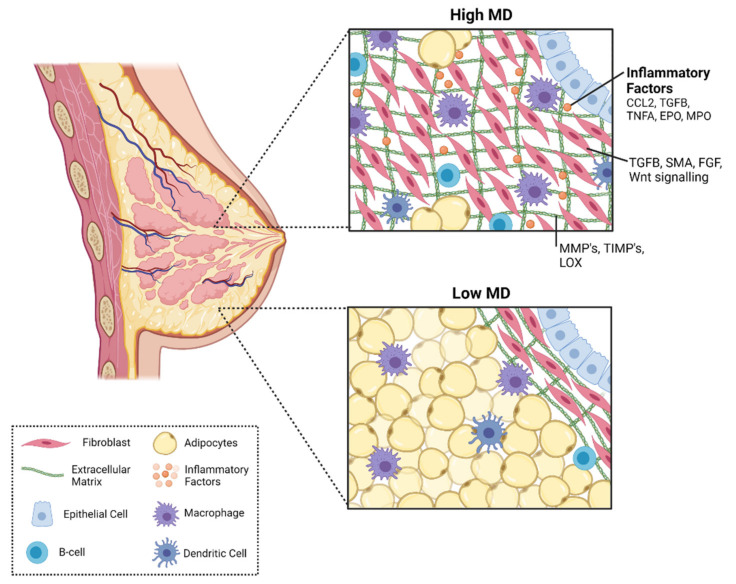
Biological mechanisms that are likely to contribute to high mammographic density. Interactions between epithelium, stroma, the extracellular matrix, and immune signalling may contribute to elevated mammographic density. Figure created using Biorender.com (Toronto, Canada).

The human breast, or mammary glands, are a unique bilateral tissue that undergoes major development during puberty, pregnancy, and during lactation [69]. Mammary glands are a defining feature of all mammals, and the primary function is to produce milk for their offspring [70]. The mammary gland is composed of a network of ducts and lobules, connected to the nipple, which expand to become milk secreting elements during lactation [71]. These structures are composed of mammary epithelial cells, which are also the cells from which most breast cancers manifest [72]. The epithelial structures are surrounded by stroma, composed of mammary fibroblasts, collagen, immune cells and extracellular matrix (ECM) [70]. The role of the mammary stroma is to provide structural and signalling support and promote appropriate development and function of the epithelium [73]. The relative abundance of epithelium, stroma and adipose tissue governs how white and dark the breast appears on an x-ray mammogram and thus is a key determinant of mammographic density. The crosstalk between cells of the stroma and other cells of the mammary gland can mediate breast cancer risk [74]. Growing evidence suggests that complex interactions between epithelium, stroma, the extracellular matrix, and immune signalling factors may contribute to elevated mammographic density (summarised in Figure 2).

### 4.1. Mammary Gland Epithelium

To understand how the mammary gland epithelium might contribute to mammographic density, a number of studies have compared histology of breast biopsies or breast tissue collected from autopsies of known mammographic density determined by the mammogram. These studies demonstrate that there is a greater abundance of epithelial cells, and epithelial cell hyperplasia in radiographically dense breast tissue [75,76,77,78]. However, many of these studies compared tissues with benign lesions, instead of normal breast tissue. More recent studies have used tissues from women undergoing prophylactic mastectomies or reduction mammoplasties to analyse the tissue composition of mammographic density. Two studies used X-ray guided biopsies to extract paired samples of high and low mammographic density from within one breast. This is a powerful tool, as it eliminates inter-patient variability providing greater statistical power. One study found no difference in glandular area between high- and low-density tissues from the same breast [79]. However, a later study with a greater sample size determined that areas of high mammographic density are composed of a greater proportion of epithelium compared to low density regions [7]. It has been supposed that mammographic density could be attributed to a greater rate of cellular proliferation, however, there is no difference in proliferation between high- and low-density tissues, as determined by staining with Ki67 [7,80,81].

### 4.2. Mammary Gland Stroma

The mammary gland stromal compartment is comprised of fibroblasts, immune cells, and ECM proteins such as collagen fibres [73]. The role of the stroma in the normal mammary gland is to provide physical support for the ductal network, and the cells of the stroma interact with the epithelium to regulate epithelial cell development and function [82]. However, these interactions can also play a role in breast cancer risk and tumour progression.

Some of the studies that investigated the abundance of mammary epithelium in mammographic density also analysed the proportions of stroma. This was analysed in multiple ways; some studies determined the proportion of stroma in breast tissues by measuring the area of non-epithelial cells and collagen [78]. More recent studies using image guided biopsy of paired high- and low-density tissues quantified stroma by calculating the percentage of stromal fibroblasts. Studies using both methods of measurement have reported that breast tissue with high density has a greater proportion of stroma, and greater percentage of stromal fibroblasts compared to breast tissue with low density [78,79,80,83]. In a study by Huo et al., it was reported that high mammographic density tissues have 30% more stroma, and 40% less adipose tissue than low mammographic density tissues. To further delineate the components of mammographic density, further studies have investigated individual components of the stroma in high- and low-density tissues [7].

Fibroblasts are the most prominent cell type within the mammary stroma. They are non-inflammatory, non-vascular, non-epithelial cells. The primary role for mammary fibroblasts is production and regulation of turnover of collagen and other components of the extracellular matrix [84]. Fibroblasts also regulate the growth and differentiation of the surrounding epithelial cells through expression of paracrine factors [85]. Fibroblasts have been well studied for their roles in cancer, where the tumour stroma is composed of cancer associated fibroblasts (CAFs). CAFs produce growth factors and cytokines that directly promote breast cancer cell proliferation, and indirectly by signalling to other cells in the microenvironment [86] and these cells could contribute to increased breast and potentially play a role in cancer risk associated with high mammographic density.

CAFs can modulate the immune microenvironment by recruiting immune cells such as M2 macrophages and T cells to dampen anti-tumour immunity by expression of chemotactic cytokines [87]. They also secrete several cytokines to sustain a chronic inflammatory environment to drive pro-tumorigenic signals such as chemokine ligand 2 (CCL2), interleukin 6 (IL6), interleukin 8 (IL8), wingless related integration (WNT) and cyclooxygenase 2 (COX2) [84,87,88]. CAFs produce high levels of collagen and ECM remodelling proteins such as tenascin-C, fibronectin and matrix metalloproteinases (MMPs) [89,90]. This creates a microenvironment that promotes tumour invasion and metastasis and is associated with reduce survival in breast cancer patients [86,91]. Though there is no standard marker of CAFs, they are generally identified by high gene expression of smooth muscle actin (SMA) and fibroblast growth factors (FGF) [92]. CAFs, or CAF-like fibroblasts, arise when fibroblasts are stimulated by activating signals from surrounding cells such as transforming growth factor beta (TGFB) and tumour necrosis factor alpha (TNFA) [93,94].

### 4.3. Collagen

The extracellular matrix and collagen are key components of the mammary stroma and are produced primarily by mammary fibroblasts [95]. ECM proteins and collagen provide structural integrity to the breast, and can have roles in breast cancer [96]. Previous studies have investigated the relationship between collagen and mammographic density using different methods. Two studies compared screening mammograms to haematoxylin and eosin-stained biopsies and determined collagen content by visual inspection by a pathologist [75,83]. More recently, studies have used image guided paired samples of high- and low-density breast tissue and Masson’s trichrome stain. This method is a more reliable measurement as it specifically stains collagen fibres, allowing for quantitative assessment of the percentage area occupied by collagen. These studies reported that high mammographic density tissues have a greater percentage of collagen compared to low mammographic density tissues [7,78]. The study by Huo et al., further analysed the collagen content in high and low mammographic density tissues by Second Harmonic Generation imaging and discovered that the collagen fibres in high mammographic density tissues are more tightly packed and have higher organisation than low mammographic density tissues [7]. Continuous activation of fibroblasts can result in excessive deposition of collagen and other ECM components to develop fibrosis [97]. This can occur under stimulation from immune cytokines such as TGFB and TNFA [98].

High abundance of collagen has been implicated in breast cancer risk. Previous studies used a transgenic mouse with increased stromal collagen in the mammary glands. These mice exhibited three times the amount of tumour formation compared to control mice, and the tumours were more invasive and had greater metastasis [99]. This study proposed that the high abundance of collagen may directly promote tumorigenesis by influencing mammary fibroblasts to secrete growth factors and cytokines to promote tumorigenesis in neighbouring epithelial cells, or directly in epithelial cells through perturbation of focal adhesion by ECM stiffness [99,100]. ECM stiffness is facilitated by lysyl oxidase (LOX), which mediates covalent cross-linking between collagen molecules to assemble collagen fibres. Expression of LOX is elevated in cancer and has been demonstrated to promote tumour growth and invasion in mouse models of breast cancer [101].

In the mammary gland, collagen is regulated by other proteins in the stroma produced by surrounding fibroblasts and immune cells [73]. Matrix metalloproteinases (MMPs) break down components of the ECM including collagen and the basement membrane, and this action is inhibited by tissue inhibitors of metalloproteinases (TIMPs) [102,103]. One study has investigated these enzymes in mammographic density; however, the associations were inconsistent and appeared to be dependent on ethnicity [104]. In breast cancer, MMPs are secreted by the tumour, surrounding fibroblasts, and infiltrating immune cells to remodel the ECM, promoting tumorigenesis, invasion, angiogenesis and metastasis [105,106].

### 4.4. Immune Cells

Immune cells are a key component of the mammary gland and are vital in facilitating mammary gland development and in immune surveillance to protect against potentially tumorigenic cells [107]. However, immune cells in the mammary gland can also contribute to breast cancer risk and progression of breast cancer. Two studies have examined the immune cell profile using paired, image guided samples of high- and low-density breast tissue to histologically stain for several immune markers. This study reported that the epithelium of high mammographic density breast tissue had a higher percentage of vimentin+/CD45+ immune cells suggestive of local inflammation in these areas [7]. Further analysis revealed that high mammographic density tissues had a greater percentage of CD4+ T cells, CD20cy+ B cells and CD11c+ dendritic cells, and no difference in populations of CD8+ cytotoxic T cells, CD56+ natural killer cells (NK), compared to low mammographic density tissues [8].

Infiltration of immune cells can influence the growth and progression of breast cancer, and can be an indicator of prognosis. Infiltration of CD8+ cytotoxic T cells and CD56+ NK cells has been observed to be favourable the patient outcomes with breast cancer, as both these cells play a role in anti-tumour immunity [108,109]. Dendritic cells, T cells and B cells are important in the normal mammary gland to maintain tumour surveillance [107]. Dendritic cells present tumour antigens to activate T cells to mediate killing of tumour cells [110]. However, depending on the immune cell profile, infiltration of immune cells in the tumour microenvironment can have both have anti-tumour effects or tumour promoting effects. Infiltration of breast cancers with T cells and B cells has been associated with increased invasiveness and poor prognosis [111,112]. It is suggested that the CD4+ T cells in high mammographic density tissues are of the Th2 subtype, as these tissues express high levels of IL-6 and IL-4 that drive differentiation to Th2 T cells [8]. These cells are associated with tumour promoting signals and are often secreted by tumours themselves to help tumour cells grow and evade immune surveillance [113].

Macrophages are highly plastic cells and key immune cells in the mammary gland. They have multiple functions in regulating mammary gland development during the ovarian cycle, pregnancy, lactation, and remodelling the mammary gland back to its pre-pregnant state [114]. They also play a role in breast cancer and can have both pro-tumorigenic and anti-tumorigenic activities. In high mammographic density tissues, there is a greater percentage of CD68+ macrophages in the epithelium, however, the phenotype and function of these cells in high mammographic density are currently unknown [8].

The function of macrophages in the mammary gland depends on their phenotype. Macrophages differentiate into different phenotypes when exposed to different cytokine signals in the microenvironment. Polarisation to classically activated macrophages, or M1 macrophages, occurs in response to infection or injury, and can be induced by exposure to microbial by-products such as lipopolysaccharide (LPS) or by cytokines interferon gamma (IFNG) and TNFA [115,116]. They have anti-microbial and anti-tumorigenic functions by secreting an array of pro-inflammatory cytokines including reactive oxygen species to induce tissue damage and impair wound healing [117,118,119,120]. M1 macrophages can be identified by expression of CD80 and nitric oxide synthase (iNOS) surface markers [115,121].

Alternatively activated, or M2 macrophages, are predominantly polarised by signals from cytokines IL-4 and IL-13 and can be identified by surface marker expression of CD163 or CD206 [122,123]. M2 macrophages are anti-inflammatory and produce abundant IL-10 and TGFB [116]. These cells are pro-tumorigenic, as they have immune suppression functions that help tumour cells evade the immune system [124]. They are also involved in tissue remodelling through production of ECM components, and enzymes such as MMPs to degrade collagen and other ECM components [119,125,126]. This helps to facilitate angiogenesis and metastasis of tumour cells along with production of pro-angiogenic factors such as vascular endothelial growth factor (VEGF) and platelet derived growth factor (PDGF) [116,127]. Polarisation to the M2 phenotype can occur during chronic inflammation [111].

Due to their involvement with tumour progression, a subset of M2 macrophages have been identified in the tumour microenvironment as tumour associated macrophages (TAMs) [115,128,129]. Similar to M2 macrophages, TAMs have high expression of IL-10, TGFB and VEGF [115,116]. Infiltration of TAMs in the tumour microenvironment is associated with a more aggressive disease, poor prognosis, and decreased survival in breast cancer patients [121,129]. Recent research is investigating the potential of repolarising M2-like TAMs towards an M1 phenotype to promote anti-tumorigenic immunity [121]. Currently, macrophage polarisation in a stroma rich environment such as high mammographic density breast tissue has not been investigated.

## 5. Immune Signaling Factors in Mammographic Density and Breast Cancer Risk

### 5.1. Monocyte Chemotactic Protein 1 (CCL2)

Monocyte chemotactic protein 1, or CCL2, is a pro-inflammatory cytokine. It is produced by many cell types including epithelial cells, tumor cells, fibroblasts, and adipocytes, as well as immune cells such as monocytes and macrophages [130,131]. The CCL2 receptor, CCR2, is predominantly expressed on monocytes and macrophages, but also other leukocytes, fibroblasts, and endothelial cells [132,133]. The primary function of CCL2 is as a chemoattractant for leukocytes, particularly monocytes and macrophages, and recruits these cells areas of tissue injury and inflammation [133].

Many studies have investigated the role of CCL2 in breast cancer and suggest that CCL2 increases breast cancer risk and tumor progression. In an *Mmtv-Ccl2* transgenic mouse model, constitutive expression of CCL2 in the mammary glands reduced tumour latency and tumor free survival when challenged with the chemical carcinogen DMBA [134]. Intravenous administration of CCL2 to mice with xenograft human breast tumours increased metastasis to the lungs and bone, and an increase in macrophage infiltration to these sites [135]. This has been further confirmed in CCL2 knockout mice in which metastasis of tumor cells was reduced [136]. In humans, expression of CCL2 is highly elevated during tumor development, and is associated with infiltration of macrophages [137,138,139]. CCL2 expression by breast tumors is associated with high tumor grade, angiogenesis, metastasis, and poor prognosis [140,141]. However, there is some evidence that CCL2 can also have some suppressive effects on tumor development through recruitment of T cells that mediate anti-tumor immunity, although these effects of CCL2 have not been studied extensively [142,143].

CCL2 has been implicated in many fibrotic diseases. CCL2 is highly expressed in the lungs of patients with lung fibrosis [144]. Animal studies have demonstrated that CCL2 deficient mice do not develop pulmonary fibrosis in response to bleomycin, a drug that induces fibrosis [145]. Bleomycin-induced fibrosis can also be reduced in mice when treated with anti-CCL2 therapies [146]. CCL2 promotes excessive production of ECM and collagen in hepatic fibrosis and renal fibrosis [147]. CCL2 can drive fibrosis by activating fibroblasts directly to exacerbate ECM and collagen production [148]. In *Mmtv-Ccl2* mice, constitutive expression of CCL2 in the mammary glands results in greater stromal thickness and collagen deposition surrounding the epithelium [134].

The roles of CCL2 in breast cancer risk and fibrosis suggest that CCL2 could promote increased mammographic density. In women, paired high and low mammographic density tissue samples revealed that CCL2 expression is elevated in the epithelium of high mammographic density breast tissue [134]. This study, along with the literature suggest that CCL2 could be an inflammatory driver of mammographic density and the associated breast cancer risk.

### 5.2. Transforming Growth Factor Beta 1 (TGFB1)

Transforming growth factor beta 1 (TGFB1) is a highly pleiotropic cytokine with a diverse number of cellular functions. It is involved in proliferation, differentiation, apoptosis, extracellular deposition, as well as modulating inflammation and immune responses [149,150]. TGFB1 is secreted by almost all cell types in the body including epithelial cells, fibroblasts, macrophages and other immune cells and its receptors are expressed on most cell types [149,151].

TGFB1 plays many roles in breast cancer, however, these functions can be both tumor suppressive and tumor promoting. The tumor suppressive functions are shown in murine studies of early tumorigenesis when constitutive expression of TGFB1 in the mammary gland results in decreased mammary tumor susceptibility when challenged with the chemical carcinogen 7,12-Dimethylbenz(a)anthracene (DMBA) [152]. When TGFB1 signaling is inhibited in the mammary gland, mice exhibit shorter tumor latency and increased tumor incidence [153]. However, this is dependent on the cell types targeted by TGFB1 deficiency; when TGFB signaling is inhibited specifically in the macrophage population, reduced susceptibility to DMBA carcinogen occurs [154]. In early cancer development, TGFB1 can disrupt the cell cycle in breast cancer cells to induce apoptosis to inhibit growth and suppress tumor development [155,156,157,158]. However, TGFB1 can also act as a tumor promotor during later stages of tumor development. TGFB1 functions to promote transformation of cells and is associated with tumor invasion [158,159,160]. Many studies have linked TGFB1 to metastasis. Mice with overexpression of TGFB1 in the mammary glands had greater tumor invasion and metastasis [161]. Further, expression of TGFB1 in breast cancers is associated with metastasis [162]. TGFB1 is a key modulator of angiogenesis, by regulating cell proliferation and migration, as well as regulation of ECM turnover and suppression of anti-tumor immunity [163,164].

TGFB1 is a key regulator of fibroblast activity and regulation of ECM deposition and turnover, and is therefore, implicated in many fibrotic diseases. Animal models have demonstrated the link between TGFB1 and fibrosis, for example overexpression of TGFB1 in rat lungs induces pulmonary fibrosis [165]. In a mouse model, constitutive expression of TGFB1 in the liver results in extensive hepatic fibrosis [166]. Inhibition of TGFB signalling in animal models of fibrosis has been shown to reduce renal, cardiac and hepatic fibrosis [167,168,169]. TGFB1 can contribute to fibrosis by stimulation of fibroblast activation through upregulation of connective tissue growth factor (CTGF) [170]. It also promotes expression of collagen genes and downregulates ECM proteinases such as MMP1 to preserve the ECM structure [171].

There have been few studies that have investigated the role of TGFB in mammographic density. ChipSeq analysis revealed several genes involved in TGFB signaling exhibited decreased expression in breast tissue of women with high mammographic density compared to those with low mammographic density [10]. Further, another study reported single nucleotide polymorphisms in TGFB1 gene which impaired TGFB signaling were associated with increased percentage mammographic density in nulliparous women [172]. The multifunctional and contradictory roles of TGFB1 in fibrotic activity in the stroma, and in both promoting and suppressing breast cancer development highlights that more investigation is required to determine the role of TGFB1 in mammographic density and breast cancer risk.

### 5.3. Peroxidase Enzymes

Myeloperoxidase (MPO), produced by monocytes, and eosinophil peroxidase (EPO), produced by eosinophils, are peroxidase enzymes released by cells during inflammation and primarily known for their role in oxidative defense against invading bacterial pathogens [173]. However, MPO and EPO can also promote fibrosis associated with inflammatory diseases. For example, MPO has been identified to have pro-fibrotic effects in the liver during non-alcoholic steatohepatitis and is found in high levels in the sputum of patients with cystic fibrosis [174,175]. EPO has been implicated in renal fibrosis and is increased in the serum of cystic fibrosis patients [176,177]. In a previous study by DeNichilo et al., the role of peroxidase enzymes in fibrotic diseases was investigated by examining the direct effects of MPO and EPO on fibroblasts from different tissue types [178]. This study found that both MPO and EPO can stimulate mammary fibroblasts to produce collagen I and IV in vitro. In breast cancer, it has previously been reported that deposits of EPO can be found within and around the tumors and play a role in HER2 positive breast cancers to promote tumor growth [179,180,181]. Several studies have reported that concentration of circulating MPO in serum is elevated in breast cancer patients [111].

### 5.4. Tumour Necrosis Factor Alpha (TNFA)

Tumour necrosis factor alpha (TNFA) is a pleiotropic cytokine involved in inflammation [182]. It is produced primarily by macrophages, but also several other cells including CD4+ T lymphocytes, mast cells, endothelial cells, adipocytes, fibroblasts and tumour cells, including breast cancer cells [183]. Studies have investigated the role of TNFA in breast cancer, however, the literature suggests that it plays opposing roles. Circulating concentration of TNFA is positively correlated with breast cancer invasiveness and poor prognosis [184,185]. In vitro studies have implicated TNFA in tumour growth, invasion, and enhanced metastasis to the lungs. In ER positive, HER2 positive, and in triple negative breast cancer cell lines, TNFA has induced proliferation, migration, and invasion [186,187]. Further, TNFA induces production of MMP9 by cancer cells that enhances tissue remodeling to promote angiogenesis and metastasis [187,188]. Conversely, TNFA can also inhibit tumor growth and kill different types of tumor cells in leukemia, lymphoma, liver cancer and breast cancer. In one study, TNFA induced cytotoxic cell death in ER positive breast cancer cell lines [189]. Its use as a targeted anti-cancer treatment is being investigated in combination with chemotherapeutics and radiation to treat breast cancer, however, some studies have reported that TNFA can promote resistance to chemotherapy in some breast cancer cells [190,191]. Clearly, the role of TNFA in breast cancer is complex, and its role in inflammation driven breast cancer risk still requires investigation.

There have been few studies investigating the role of TNFA in mammographic density. One study measured the relationship between circulating inflammatory markers in relation to mammographic density obtained from screening mammograms, this study found no association between serum TNFA concentration and mammographic density [192]. However, another study measured breast tissue gene expression of TNFA in post-menopausal women and found that high mammographic density was associated with higher expression of TNFA [193]. The role of TNFA in mammographic density is currently unknown, however it could regulate mammary fibroblast activity and fibrosis. Studies of TNFA have revealed that it can have both pro and anti-fibrotic effects in models of fibrotic diseases [194]. TNFA promotes proliferation of fibroblasts, increases production of tissue remodelling enzymes including MMPs [195]. In diseases characterised by fibrosis, such as Crohn’s disease, TNFA has been shown to increase collagen accumulation [196]. Conversely, other studies have reported that TNFA can inhibit production of collagen I in cultured fibroblasts [197]. The role of TNFA in mammographic density and mammary fibroblast activity appear to be complex and requires investigation.

## 6. Inflammation as a Target for Therapeutic Intervention

Chronic inflammation is one of the hallmarks of cancer, and can promote mammary tumour initiation, growth, invasion, and metastasis. Elevated markers of inflammation such as C-reactive protein (CRP), IL-6 and TNFA are associated with an increased risk of breast cancer, increased risk of recurrence, and reduced tumour free survival [198]. The use of non-steroidal anti-inflammatory drugs (NSAIDs) can be employed as a preventative agent that reduces the risk of many different types of cancer [199,200]. Daily use of NSAIDs, particularly aspirin, can reduce risk of breast cancer by up to 39% [200].

Studies have implicated inflammation as a driver of mammographic density. In a study that compared mammograms to healthy breast tissue taken from breast surgeries, it was reported that COX2 expression was greater in the stroma of high mammographic density breasts compared to low mammographic density breasts [10]. This was confirmed in a study of image guided paired sampling of high and low mammographic density tissues from within one breast that revealed a greater abundance of COX2 in the epithelium and stroma of high mammographic density, compared to low mammographic density tissues [201]. Further, paired high and low mammographic density tissue analysis has demonstrated that high mammographic density tissues have higher expression of IL6 and IL4, and increased infiltration of vimentin-positive immune cells than low mammographic density tissue [8]. Genome wide studies have detected genetic variations in the IL-6 gene are associated with high mammographic density [202] indicative of an inflammatory microenvironment in high mammographic density breast tissue. In a mouse model with high abundance of collagen in the mammary glands, reminiscent of mammographic density, treatment with a COX2 inhibitor reduced tumor size and metastasis [203].

As pharmaceutical inhibitors of COX2, NSAID use has the potential to decrease breast cancer risk in women with high mammographic density. It is currently unclear whether NSAID use reduces mammographic density; a randomized controlled trial suggested aspirin (325 mg/day) was not effective in lowering mammographic density [204], however, the study was in a small sample size and of short duration (*n* = 143; 6 months). Large cohort studies have found promising associations between NSAID use and lower mammographic density, although not all studies have found an association [205,206,207]. Nonetheless, it is critical that studies on therapeutic interventions should primarily focus on reduction of risk of breast cancer associated with high mammographic density, not mammographic density per se. To use reduced mammographic density as a surrogate biomarker for reduced breast cancer risk is to assume a causal biological relationship [208], and currently these biological pathways are still under investigation.

## 7. Conclusions

Mammographic density is strong independent risk factor for breast cancer. Women with high mammographic density have a four to six fold increased risk of breast cancer compared to women with low mammographic density when matched for age and BMI. Mammographic density is influenced by age, hereditary factors, BMI, parity, menopausal status, and hormonal therapies. Histological studies have revealed that high mammographic density is characterised by high proportions of stroma containing fibroblasts and an immune cell profile indicative of an inflammatory, tumour promoting environment. However, the underlying biological mechanisms that mediate mammographic density are yet to be elucidated.

Chronic inflammation has been established to contribute to breast cancer risk and development. There are number of immune and inflammatory factors that contribute to breast cancer risk, as well influence the stromal environment. Peroxidase enzymes, TGFB, TNFA and CCL2 have been demonstrated to influence the stromal environment through effects on fibroblast activity and immune cells, as well as contributing to breast cancer risk and development. Of particular therapeutic interest, anti-inflammatory drugs hold promise in reducing mammographic density-associated breast cancer risk and further large-scale studies could provide new options for primary prevention. However, further research on the immune signaling factors active in breast tissue are needed to establish causal biological pathways. Understanding the drivers of mammographic density and the associated increased breast cancer risk may provide targets for preventative therapies in the future for women with high mammographic density.

## References

[1] Heer E., Harper A., Escandor N., Sung H., McCormack V., Fidler-Benaoudia M.M. (2020). Global burden and trends in premenopausal and postmenopausal breast cancer: A population-based study. Lancet Glob. Health.

[2] Kotsopoulos J. (2018). BRCA Mutations and Breast Cancer Prevention. Cancers.

[3] Melvin J.C., Wulaningsih W., Hana Z., Purushotham A.D., Pinder S.E., Fentiman I., Gillett C., Mera A., Holmberg L., Van Hemelrijck M. (2016). Family history of breast cancer and its association with disease severity and mortality. Cancer Med..

[4] Ziegler R.G., Hoover R.N., Pike M.C., Hildesheim A., Nomura A.M., West D.W., Wu-Williams A.H., Kolonel L.N., Horn-Ross P.L., Rosenthal J.F. (1993). Migration patterns and breast cancer risk in Asian-American women. J. Nat. Cancer Inst..

[5] Boyd N.F., Guo H., Martin L.J., Sun L., Stone J., Fishell E., Jong R.A., Hislop G., Chiarelli A., Minkin S. (2007). Mammographic density and the risk and detection of breast cancer. N. Engl. J. Med..

[6] Engmann N.J., Golmakani M.K., Miglioretti D.L., Sprague B.L., Kerlikowske K. (2017). Population-Attributable Risk Proportion of Clinical Risk Factors for Breast Cancer. JAMA Oncol..

[7] Huo C.W., Chew G., Hill P., Huang D., Ingman W., Hodson L., Brown K.A., Magenau A., Allam A.H., McGhee E. (2015). High mammographic density is associated with an increase in stromal collagen and immune cells within the mammary epithelium. Breast Cancer Res..

[8] Huo C.W., Hill P., Chew G., Neeson P.J., Halse H., Williams E.D., Henderson M.A., Thompson E.W., Britt K.L. (2018). High mammographic density in women is associated with protumor inflammation. Breast Cancer Res..

[9] Woolcott C.G., Courneya K.S., Boyd N.F., Yaffe M.J., McTiernan A., Brant R., Jones C.A., Stanczyk F.Z., Terry T., Cook L.S. (2013). Association between sex hormones, glucose homeostasis, adipokines, and inflammatory markers and mammographic density among postmenopausal women. Breast Cancer Res. Treat..

[10] Yang W.T., Lewis M.T., Hess K., Wong H., Tsimelzon A., Karadag N., Cairo M., Wei C., Meric-Bernstam F., Brown P. (2010). Decreased TGFbeta signaling and increased COX2 expression in high risk women with increased mammographic breast density. Breast Cancer Res. Treat..

[11] Johns P.C., Yaffe M.J. (1987). X-ray characterisation of normal and neoplastic breast tissues. Phys. Med. Biol..

[12] Hugo H.J., Zysk A., Dasari P., Britt K., Hopper J.L., Stone J., Thompson E.W., Ingman W.V. (2018). InforMD: A new initiative to raise public awareness about breast density. Ecancermedicalscience.

[13] Boyd N.F. (2013). Mammographic density and risk of breast cancer. Am. Soc. Clin. Oncol. Educ. Book.

[14] Ghadge A.G., Dasari P., Stone J., Thompson E.W., Robker R.L., Ingman W.V. (2021). Pubertal mammary gland development is a key determinant of adult mammographic density. Semin. Cell Dev. Biol..

[15] Timmers J.M., van Doorne-Nagtegaal H.J., Zonderland H.M., van Tinteren H., Visser O., Verbeek A.L., den Heeten G.J., Broeders M.J. (2012). The Breast Imaging Reporting and Data System (BI-RADS) in the Dutch breast cancer screening programme: Its role as an assessment and stratification tool. Eur. Radiol..

[16] Sprague B.L., Gangnon R.E., Burt V., Trentham-Dietz A., Hampton J.M., Wellman R.D., Kerlikowske K., Miglioretti D.L. (2014). Prevalence of mammographically dense breasts in the United States. J. Nat. Cancer Inst..

[17] Jeffers A.M., Sieh W., Lipson J.A., Rothstein J.H., McGuire V., Whittemore A.S., Rubin D.L. (2017). Breast Cancer Risk and Mammographic Density Assessed with Semiautomated and Fully Automated Methods and BI-RADS. Radiology.

[18] Astley S.M., Harkness E.F., Sergeant J.C., Warwick J., Stavrinos P., Warren R., Wilson M., Beetles U., Gadde S., Lim Y. (2018). A comparison of five methods of measuring mammographic density: A case-control study. Breast Cancer Res..

[19] Wolfe J.N. (1976). Risk for breast cancer development determined by mammographic parenchymal pattern. Cancer.

[20] Boyd N.F., Martin L.J., Sun L., Guo H., Chiarelli A., Hislop G., Yaffe M., Minkin S. (2006). Body size, mammographic density, and breast cancer risk. Cancer Epidemiol. Biomark. Prev..

[21] McCormack V.A., dos Santos Silva I. (2006). Breast density and parenchymal patterns as markers of breast cancer risk: A meta-analysis. Cancer Epidemiol. Biomark. Prev..

[22] Vachon C.M., Brandt K.R., Ghosh K., Scott C.G., Maloney S.D., Carston M.J., Pankratz V.S., Sellers T.A. (2007). Mammographic breast density as a general marker of breast cancer risk. Cancer Epidemiol. Biomark. Prev..

[23] Eriksson L., Hall P., Czene K., Dos Santos Silva I., McCormack V., Bergh J., Bjohle J., Ploner A. (2012). Mammographic density and molecular subtypes of breast cancer. Br. J. Cancer.

[24] Yaghjyan L., Colditz G.A., Collins L.C., Schnitt S.J., Rosner B., Vachon C., Tamimi R.M. (2011). Mammographic breast density and subsequent risk of breast cancer in postmenopausal women according to tumor characteristics. J. Nat. Cancer Inst..

[25] Heusinger K., Jud S.M., Haberle L., Hack C.C., Adamietz B.R., Meier-Meitinger M., Lux M.P., Wittenberg T., Wagner F., Loehberg C.R. (2012). Association of mammographic density with hormone receptors in invasive breast cancers: Results from a case-only study. Int. J. Cancer.

[26] Ding J., Warren R., Girling A., Thompson D., Easton D. (2010). Mammographic density, estrogen receptor status and other breast cancer tumor characteristics. Breast J..

[27] Mokhtary A., Karakatsanis A., Valachis A. (2021). Mammographic Density Changes over Time and Breast Cancer Risk: A Systematic Review and Meta-Analysis. Cancers.

[28] Bertrand K.A., Tamimi R.M., Scott C.G., Jensen M.R., Pankratz V., Visscher D., Norman A., Couch F., Shepherd J., Fan B. (2013). Mammographic density and risk of breast cancer by age and tumor characteristics. Breast Cancer Res..

[29] Eriksson L., Czene K., Rosenberg L., Humphreys K., Hall P. (2013). Possible influence of mammographic density on local and locoregional recurrence of breast cancer. Breast Cancer Res..

[30] Maskarinec G., Pagano I.S., Little M.A., Conroy S.M., Park S.Y., Kolonel L.N. (2013). Mammographic density as a predictor of breast cancer survival: The Multiethnic Cohort. Breast Cancer Res..

[31] Gierach G.L., Ichikawa L., Kerlikowske K., Brinton L.A., Farhat G.N., Vacek P.M., Weaver D.L., Schairer C., Taplin S.H., Sherman M.E. (2012). Relationship between mammographic density and breast cancer death in the Breast Cancer Surveillance Consortium. J. Nat. Cancer Inst..

[32] Chiu S.Y., Duffy S., Yen A.M., Tabar L., Smith R.A., Chen H.H. (2010). Effect of baseline breast density on breast cancer incidence, stage, mortality, and screening parameters: 25-year follow-up of a Swedish mammographic screening. Cancer Epidemiol. Biomark. Prev..

[33] Weigel S., Heindel W., Heidrich J., Hense H.W., Heidinger O. (2017). Digital mammography screening: Sensitivity of the programme dependent on breast density. Eur. Radiol..

[34] Sandberg M.E., Li J., Hall P., Hartman M., dos-Santos-Silva I., Humphreys K., Czene K. (2013). Change of mammographic density predicts the risk of contralateral breast cancer--a case-control study. Breast Cancer Res..

[35] Cil T., Fishell E., Hanna W., Sun P., Rawlinson E., Narod S.A., McCready D.R. (2009). Mammographic density and the risk of breast cancer recurrence after breast-conserving surgery. Cancer.

[36] Buist D.S., Abraham L.A., Barlow W.E., Krishnaraj A., Holdridge R.C., Sickles E.A., Carney P.A., Kerlikowske K., Geller B.M. (2010). Diagnosis of second breast cancer events after initial diagnosis of early stage breast cancer. Breast Cancer Res. Treat..

[37] Sung H., Ren J., Li J., Pfeiffer R.M., Wang Y., Guida J.L., Fang Y., Shi J., Zhang K., Li N. (2018). Breast cancer risk factors and mammographic density among high-risk women in urban China. NPJ Breast Cancer.

[38] Moore J.X., Han Y., Appleton C., Colditz G., Toriola A.T. (2020). Determinants of Mammographic Breast Density by Race Among a Large Screening Population. JNCI Cancer Spectr..

[39] Gierach G.L., Loud J.T., Chow C.K., Prindiville S.A., Eng-Wong J., Soballe P.W., Giambartolomei C., Mai P.L., Galbo C.E., Nichols K. (2010). Mammographic density does not differ between unaffected BRCA1/2 mutation carriers and women at low-to-average risk of breast cancer. Breast Cancer Res. Treat..

[40] Ramon Y.C.T., Chirivella I., Miranda J., Teule A., Izquierdo A., Balmana J., Sanchez-Heras A.B., Llort G., Fisas D., Lope V. (2015). Mammographic density and breast cancer in women from high risk families. Breast Cancer Res..

[41] Pankow J.S., Vachon C.M., Kuni C.C., King R.A., Arnett D.K., Grabrick D.M., Rich S.S., Anderson V.E., Sellers T.A. (1997). Genetic analysis of mammographic breast density in adult women: Evidence of a gene effect. J. Nat. Cancer Inst..

[42] Boyd N.F., Dite G.S., Stone J., Gunasekara A., English D.R., McCredie M.R., Giles G.G., Tritchler D., Chiarelli A., Yaffe M.J. (2002). Heritability of mammographic density, a risk factor for breast cancer. N. Engl. J. Med..

[43] Reeves K.W., Stone R.A., Modugno F., Ness R.B., Vogel V.G., Weissfeld J.L., Habel L.A., Sternfeld B., Cauley J.A. (2009). Longitudinal association of anthropometry with mammographic breast density in the Study of Women’s Health Across the Nation. Int. J. Cancer.

[44] Tamimi R.M., Byrne C., Colditz G.A., Hankinson S.E. (2007). Endogenous hormone levels, mammographic density, and subsequent risk of breast cancer in postmenopausal women. J. Nat. Cancer Inst..

[45] Boyd N.F., Lockwood G.A., Byng J.W., Little L.E., Yaffe M.J., Tritchler D.L. (1998). The relationship of anthropometric measures to radiological features of the breast in premenopausal women. Br. J. Cancer.

[46] Hjerkind K.V., Ellingjord-Dale M., Johansson A.L.V., Aase H.S., Hoff S.R., Hofvind S., Fagerheim S., Dos-Santos-Silva I., Ursin G. (2018). Volumetric Mammographic Density, Age-Related Decline, and Breast Cancer Risk Factors in a National Breast Cancer Screening Program. Cancer Epidemiol. Biomark. Prev..

[47] Checka C.M., Chun J.E., Schnabel F.R., Lee J., Toth H. (2012). The relationship of mammographic density and age: Implications for breast cancer screening. AJR Am. J. Roentgenol..

[48] Burton A., Maskarinec G., Perez-Gomez B., Vachon C., Miao H., Lajous M., López-Ridaura R., Rice M., Pereira A., Garmendia M.L. (2017). Mammographic density and ageing: A collaborative pooled analysis of cross-sectional data from 22 countries worldwide. PLOS Med..

[49] Australian Institute of Health and Welfare (2012). Breast Cancer in Australia: An Overview. Canberra AIHW.

[50] Nguyen T.L., Schmidt D.F., Makalic E., Dite G.S., Stone J., Apicella C., Bui M., Macinnis R.J., Odefrey F., Cawson J.N. (2013). Explaining variance in the cumulus mammographic measures that predict breast cancer risk: A twins and sisters study. Cancer Epidemiol. Biomark. Prev..

[51] Woolcott C.G., Koga K., Conroy S.M., Byrne C., Nagata C., Ursin G., Vachon C.M., Yaffe M.J., Pagano I., Maskarinec G. (2012). Mammographic density, parity and age at first birth, and risk of breast cancer: An analysis of four case-control studies. Breast Cancer Res. Treat..

[52] Lope V., Pérez-Gómez B., Sánchez-Contador C., Santamariña M.C., Moreo P., Vidal C., Laso M.S., Ederra M., Pedraz-Pingarrón C., González-Román I. (2012). Obstetric history and mammographic density: A population-based cross-sectional study in Spain (DDM-Spain). Breast Cancer Res. Treat..

[53] Bremnes Y., Ursin G., Bjurstam N., Lund E., Gram I.T. (2007). Different types of postmenopausal hormone therapy and mammographic density in Norwegian women. Int. J. Cancer.

[54] Couto E., Qureshi S.A., Hofvind S., Hilsen M., Aase H., Skaane P., Vatten L., Ursin G. (2012). Hormone therapy use and mammographic density in postmenopausal Norwegian women. Breast Cancer Res. Treat..

[55] Lowry S.J., Aiello Bowles E.J., Anderson M.L., Buist D.S. (2011). Predictors of breast density change after hormone therapy cessation: Results from a randomized trial. Cancer Epidemiol. Biomark. Prev..

[56] Azam S., Lange T., Huynh S., Aro A.R., von Euler-Chelpin M., Vejborg I., Tjonneland A., Lynge E., Andersen Z.J. (2018). Hormone replacement therapy, mammographic density, and breast cancer risk: A cohort study. Cancer Causes Control..

[57] Kerlikowske K., Cook A.J., Buist D.S., Cummings S.R., Vachon C., Vacek P., Miglioretti D.L. (2010). Breast cancer risk by breast density, menopause, and postmenopausal hormone therapy use. J. Clin. Oncol..

[58] Van Duijnhoven F.J., Peeters P.H., Warren R.M., Bingham S.A., van Noord P.A., Monninkhof E.M., Grobbee D.E., van Gils C.H. (2007). Postmenopausal hormone therapy and changes in mammographic density. J. Clin. Oncol..

[59] Cecchini R.S., Costantino J.P., Cauley J.A., Cronin W.M., Wickerham D.L., Bandos H., Weissfeld J.L., Wolmark N. (2012). Baseline mammographic breast density and the risk of invasive breast cancer in postmenopausal women participating in the NSABP study of tamoxifen and raloxifene (STAR). Cancer Prev. Res..

[60] Cuzick J., Warwick J., Pinney E., Duffy S.W., Cawthorn S., Howell A., Forbes J.F., Warren R.M. (2011). Tamoxifen-induced reduction in mammographic density and breast cancer risk reduction: A nested case-control study. J. Nat. Cancer Inst..

[61] Cuzick J., Warwick J., Pinney E., Warren R.M., Duffy S.W. (2004). Tamoxifen and breast density in women at increased risk of breast cancer. J. Nat. Cancer Inst..

[62] Chow C.K., Venzon D., Jones E.C., Premkumar A., O’Shaughnessy J., Zujewski J. (2000). Effect of tamoxifen on mammographic density. Cancer Epidemiol. Biomark. Prev..

[63] Li J., Humphreys K., Eriksson L., Edgren G., Czene K., Hall P. (2013). Mammographic density reduction is a prognostic marker of response to adjuvant tamoxifen therapy in postmenopausal patients with breast cancer. J. Clin. Oncol..

[64] Henry N.L., Chan H.P., Dantzer J., Goswami C.P., Li L., Skaar T.C., Rae J.M., Desta Z., Khouri N., Pinsky R. (2013). Aromatase inhibitor-induced modulation of breast density: Clinical and genetic effects. Br. J. Cancer.

[65] Kim J., Han W., Moon H.G., Ahn S., Shin H.C., You J.M., Han S.W., Im S.A., Kim T.Y., Koo H. (2012). Breast density change as a predictive surrogate for response to adjuvant endocrine therapy in hormone receptor positive breast cancer. Breast Cancer Res..

[66] Vachon C.M., Suman V.J., Brandt K.R., Kosel M.L., Buzdar A.U., Olson J.E., Wu F.F., Flickinger L.M., Ursin G., Elliott C.R. (2013). Mammographic Breast Density Response to Aromatase Inhibition. Clin. Cancer Res..

[67] Azam S., Sjolander A., Eriksson M., Gabrielson M., Czene K., Hall P. (2019). Determinants of Mammographic Density Change. JNCI Cancer Spectr..

[68] Johansson H., Gandini S., Bonanni B., Mariette F., Guerrieri-Gonzaga A., Serrano D., Cassano E., Ramazzotto F., Baglietto L., Sandri M.T. (2008). Relationships between circulating hormone levels, mammographic percent density and breast cancer risk factors in postmenopausal women. Breast Cancer Res. Treat..

[69] Richert M.M., Schwertfeger K.L., Ryder J.W., Anderson S.M. (2000). An atlas of mouse mammary gland development. J. Mammary Gland Biol. Neoplasia.

[70] Russo J., Hu Y.-F., Yang X., Russo I.H. (2000). Chapter 1: Developmental, Cellular, and Molecular Basis of Human Breast Cancer. JNCI Monogr..

[71] Hovey R.C., Trott J.F., Vonderhaar B.K. (2002). Establishing a framework for the functional mammary gland: From endocrinology to morphology. J. Mammary Gland Biol. Neoplasia.

[72] Polyak K., Kalluri R. (2010). The role of the microenvironment in mammary gland development and cancer. Cold Spring Harb. Perspect. Biol..

[73] Wiseman B.S., Werb Z. (2002). Stromal effects on mammary gland development and breast cancer. Science.

[74] Roozendaal R., Mebius R.E. (2011). Stromal cell-immune cell interactions. Annu. Rev. Immunol..

[75] Boyd N.F., Jensen H.M., Cooke G., Han H.L. (1992). Relationship between mammographic and histological risk factors for breast cancer. J. Nat. Cancer Inst..

[76] Bright R.A., Morrison A.S., Brisson J., Burstein N.A., Sadowsky N.S., Kopans D.B., Meyer J.E. (1988). Relationship between mammographic and histologic features of breast tissue in women with benign biopsies. Cancer.

[77] Bartow S.A., Pathak D.R., Mettler F.A., Key C.R., Pike M.C. (1995). Breast Mammographic Pattern: A Concatenation of Confounding and Breast Cancer Risk Factors. Am. J. Epidemiol..

[78] Li T., Sun L., Miller N., Nicklee T., Woo J., Hulse-Smith L., Tsao M.S., Khokha R., Martin L., Boyd N. (2005). The association of measured breast tissue characteristics with mammographic density and other risk factors for breast cancer. Cancer Epidemiol. Biomark. Prev..

[79] Lin S.J., Cawson J., Hill P., Haviv I., Jenkins M., Hopper J.L., Southey M.C., Campbell I.G., Thompson E.W. (2011). Image-guided sampling reveals increased stroma and lower glandular complexity in mammographically dense breast tissue. Breast Cancer Res. Treat..

[80] Ghosh K., Brandt K.R., Reynolds C., Scott C.G., Pankratz V.S., Riehle D.L., Lingle W.L., Odogwu T., Radisky D.C., Visscher D.W. (2012). Tissue composition of mammographically dense and non-dense breast tissue. Breast Cancer Res. Treat..

[81] Khan Q.J., Kimler B.F., O’Dea A.P., Zalles C.M., Sharma P., Fabian C.J. (2007). Mammographic density does not correlate with Ki-67 expression or cytomorphology in benign breast cells obtained by random periareolar fine needle aspiration from women at high risk for breast cancer. Breast Cancer Res..

[82] Conklin M.W., Keely P.J. (2012). Why the stroma matters in breast cancer: Insights into breast cancer patient outcomes through the examination of stromal biomarkers. Cell Adh. Migr..

[83] Alowami S., Troup S., Al-Haddad S., Kirkpatrick I., Watson P.H. (2003). Mammographic density is related to stroma and stromal proteoglycan expression. Breast Cancer Res..

[84] Kalluri R., Zeisberg M. (2006). Fibroblasts in cancer. Nat. Rev. Cancer.

[85] Lühr I., Friedl A., Overath T., Tholey A., Kunze T., Hilpert F., Sebens S., Arnold N., Rösel F., Oberg H.H. (2012). Mammary fibroblasts regulate morphogenesis of normal and tumorigenic breast epithelial cells by mechanical and paracrine signals. Cancer Lett..

[86] Houthuijzen J.M., Jonkers J. (2018). Cancer-associated fibroblasts as key regulators of the breast cancer tumor microenvironment. Cancer Metastasis Rev..

[87] Raz Y., Erez N. (2013). An inflammatory vicious cycle: Fibroblasts and immune cell recruitment in cancer. Exp. Cell Res..

[88] Han Y., Zhang Y., Jia T., Sun Y. (2015). Molecular mechanism underlying the tumor-promoting functions of carcinoma-associated fibroblasts. Tumour Biol..

[89] De Wever O., Nguyen Q.D., Van Hoorde L., Bracke M., Bruyneel E., Gespach C., Mareel M. (2004). Tenascin-C and SF/HGF produced by myofibroblasts in vitro provide convergent pro-invasive signals to human colon cancer cells through RhoA and Rac. FASEB J..

[90] O’Connell J.T., Sugimoto H., Cooke V.G., MacDonald B.A., Mehta A.I., LeBleu V.S., Dewar R., Rocha R.M., Brentani R.R., Resnick M.B. (2011). VEGF-A and Tenascin-C produced by S100A4+ stromal cells are important for metastatic colonization. Proc. Natl. Acad. Sci. USA.

[91] Olsen C.J., Moreira J., Lukanidin E.M., Ambartsumian N.S. (2010). Human mammary fibroblasts stimulate invasion of breast cancer cells in a three-dimensional culture and increase stroma development in mouse xenografts. BMC Cancer.

[92] Hemalatha S.K., Sengodan S.K., Nadhan R., Dev J., Sushama R.R., Somasundaram V., Thankappan R., Rajan A., Latha N.R., Varghese G.R. (2018). Brcal Defective Breast Cancer Cells Induce in vitro Transformation of Cancer Associated Fibroblasts (CAFs) to Metastasis Associated Fibroblasts (MAF). Sci. Rep..

[93] Calon A., Tauriello D.V., Batlle E. (2014). TGF-beta in CAF-mediated tumor growth and metastasis. Semin. Cancer Biol..

[94] Rasanen K., Vaheri A. (2010). Activation of fibroblasts in cancer stroma. Exp. Cell Res..

[95] Last J.A., Reiser K.M. (1984). Collagen biosynthesis. Environ. Health Perspect..

[96] Insua-Rodriguez J., Oskarsson T. (2016). The extracellular matrix in breast cancer. Adv. Drug Deliv. Rev..

[97] Uitto J., Kouba D. (2000). Cytokine modulation of extracellular matrix gene expression: Relevance to fibrotic skin diseases. J. Dermatol. Sci..

[98] Verrecchia F., Mauviel A. (2004). TGF-beta and TNF-alpha: Antagonistic cytokines controlling type I collagen gene expression. Cell Signal..

[99] Provenzano P.P., Inman D.R., Eliceiri K.W., Knittel J.G., Yan L., Rueden C.T., White J.G., Keely P.J. (2008). Collagen density promotes mammary tumor initiation and progression. BMC Med..

[100] Ghajar C.M., Bissell M.J. (2008). Extracellular matrix control of mammary gland morphogenesis and tumorigenesis: Insights from imaging. Histochem. Cell Biol..

[101] Levental K.R., Yu H., Kass L., Lakins J.N., Egeblad M., Erler J.T., Fong S.F., Csiszar K., Giaccia A., Weninger W. (2009). Matrix crosslinking forces tumor progression by enhancing integrin signaling. Cell.

[102] Chambers A.F., Matrisian L.M. (1997). Changing views of the role of matrix metalloproteinases in metastasis. J. Nat. Cancer Inst..

[103] Cheng G., Fan X., Hao M., Wang J., Zhou X., Sun X. (2016). Higher levels of TIMP-1 expression are associated with a poor prognosis in triple-negative breast cancer. Mol. Cancer.

[104] Steude J.S., Maskarinec G., Erber E., Verheus M., Hernandez B.Y., Killeen J., Cline J.M. (2010). Mammographic density and matrix metalloproteinases in breast tissue. Cancer Microenviron..

[105] Radisky E.S., Radisky D.C. (2015). Matrix metalloproteinases as breast cancer drivers and therapeutic targets. Front. Biosci..

[106] Duffy M.J., Maguire T.M., Hill A., McDermott E., O’Higgins N. (2000). Metalloproteinases: Role in breast carcinogenesis, invasion and metastasis. Breast Cancer Res..

[107] Bates J.P., Derakhshandeh R., Jones L., Webb T.J. (2018). Mechanisms of immune evasion in breast cancer. BMC Cancer.

[108] Mahmoud S.M., Paish E.C., Powe D.G., Macmillan R.D., Grainge M.J., Lee A.H., Ellis I.O., Green A.R. (2011). Tumor-infiltrating CD8+ lymphocytes predict clinical outcome in breast cancer. J. Clin. Oncol..

[109] Mamessier E., Pradel L.C., Thibult M.L., Drevet C., Zouine A., Jacquemier J., Houvenaeghel G., Bertucci F., Birnbaum D., Olive D. (2013). Peripheral blood NK cells from breast cancer patients are tumor-induced composite subsets. J. Immunol..

[110] Iwamoto M., Shinohara H., Miyamoto A., Okuzawa M., Mabuchi H., Nohara T., Gon G., Toyoda M., Tanigawa N. (2003). Prognostic value of tumor-infiltrating dendritic cells expressing CD83 in human breast carcinomas. Int. J. Cancer.

[111] DeNardo D.G., Coussens L.M. (2007). Inflammation and breast cancer. Balancing immune response: Crosstalk between adaptive and innate immune cells during breast cancer progression. Breast Cancer Res..

[112] Hussein M.R., Hassan H.I. (2006). Analysis of the mononuclear inflammatory cell infiltrate in the normal breast, benign proliferative breast disease, in situ and infiltrating ductal breast carcinomas: Preliminary observations. J. Clin. Pathol..

[113] Goto S., Sato M., Kaneko R., Itoh M., Sato S., Takeuchi S. (1999). Analysis of Th1 and Th2 cytokine production by peripheral blood mononuclear cells as a parameter of immunological dysfunction in advanced cancer patients. Cancer Immunol. Immunother..

[114] Chua A.C., Hodson L.J., Moldenhauer L.M., Robertson S.A., Ingman W.V. (2010). Dual roles for macrophages in ovarian cycle-associated development and remodelling of the mammary gland epithelium. Development.

[115] Shapouri-Moghaddam A., Mohammadian S., Vazini H., Taghadosi M., Esmaeili S.A., Mardani F., Seifi B., Mohammadi A., Afshari J.T., Sahebkar A. (2018). Macrophage plasticity, polarization, and function in health and disease. J. Cell Physiol..

[116] Jetten N., Verbruggen S., Gijbels M.J., Post M.J., De Winther M.P., Donners M.M. (2014). Anti-inflammatory M2, but not pro-inflammatory M1 macrophages promote angiogenesis in vivo. Angiogenesis.

[117] Bashir S., Sharma Y., Elahi A., Khan F. (2016). Macrophage polarization: The link between inflammation and related diseases. Inflamm. Res..

[118] Locati M., Mantovani A., Sica A. (2013). Macrophage activation and polarization as an adaptive component of innate immunity. Adv. Immunol..

[119] Mantovani A., Biswas S.K., Galdiero M.R., Sica A., Locati M. (2013). Macrophage plasticity and polarization in tissue repair and remodelling. J. Pathol..

[120] Chen K., Lu P., Beeraka N.M., Sukocheva O.A., Madhunapantula S.V., Liu J., Sinelnikov M.Y., Nikolenko V.N., Bulygin K.V., Mikhaleva L.M. (2020). Mitochondrial mutations and mitoepigenetics: Focus on regulation of oxidative stress-induced responses in breast cancers. Semin. Cancer Biol..

[121] Mukhtar R.A., Nseyo O., Campbell M.J., Esserman L.J. (2011). Tumor-associated macrophages in breast cancer as potential biomarkers for new treatments and diagnostics. Expert Rev. Mol. Diagn..

[122] Wang N., Liang H., Zen K. (2014). Molecular mechanisms that influence the macrophage m1–m2 polarization balance. Front. Immunol..

[123] Porta C., Riboldi E., Ippolito A., Sica A. (2015). Molecular and epigenetic basis of macrophage polarized activation. Semin. Immunol..

[124] Roszer T. (2015). Understanding the Mysterious M2 Macrophage through Activation Markers and Effector Mechanisms. Mediat. Inflamm..

[125] Braga T.T., Agudelo J.S., Camara N.O. (2015). Macrophages During the Fibrotic Process: M2 as Friend and Foe. Front. Immunol..

[126] Sicari B.M., Dziki J.L., Siu B.F., Medberry C.J., Dearth C.L., Badylak S.F. (2014). The promotion of a constructive macrophage phenotype by solubilized extracellular matrix. Biomaterials.

[127] Mantovani A., Sozzani S., Locati M., Allavena P., Sica A. (2002). Macrophage polarization: Tumor-associated macrophages as a paradigm for polarized M2 mononuclear phagocytes. Trends Immunol..

[128] Allavena P., Sica A., Garlanda C., Mantovani A. (2008). The Yin-Yang of tumor-associated macrophages in neoplastic progression and immune surveillance. Immunol. Rev..

[129] Sica A., Schioppa T., Mantovani A., Allavena P. (2006). Tumour-associated macrophages are a distinct M2 polarised population promoting tumour progression: Potential targets of anti-cancer therapy. Eur. J. Cancer.

[130] Cushing S.D., Berliner J.A., Valente A.J., Territo M.C., Navab M., Parhami F., Gerrity R., Schwartz C.J., Fogelman A.M. (1990). Minimally modified low density lipoprotein induces monocyte chemotactic protein 1 in human endothelial cells and smooth muscle cells. Proc. Natl. Acad. Sci. USA.

[131] Standiford T.J., Kunkel S.L., Phan S.H., Rollins B.J., Strieter R.M. (1991). Alveolar macrophage-derived cytokines induce monocyte chemoattractant protein-1 expression from human pulmonary type II-like epithelial cells. J. Biol. Chem..

[132] Carulli M.T., Ong V.H., Ponticos M., Shiwen X., Abraham D.J., Black C.M., Denton C.P. (2005). Chemokine receptor CCR2 expression by systemic sclerosis fibroblasts: Evidence for autocrine regulation of myofibroblast differentiation. Arthritis Rheum..

[133] Deshmane S.L., Kremlev S., Amini S., Sawaya B.E. (2009). Monocyte chemoattractant protein-1 (MCP-1): An overview. J. Interferon. Cytokine Res..

[134] Sun X., Glynn D.J., Hodson L.J., Huo C., Britt K., Thompson E.W., Woolford L., Evdokiou A., Pollard J.W., Robertson S.A. (2017). CCL2-driven inflammation increases mammary gland stromal density and cancer susceptibility in a transgenic mouse model. Breast Cancer Res..

[135] Lu X., Kang Y. (2009). Chemokine (C-C motif) ligand 2 engages CCR2+ stromal cells of monocytic origin to promote breast cancer metastasis to lung and bone. J. Biol. Chem..

[136] Yoshimura T., Howard O.M., Ito T., Kuwabara M., Matsukawa A., Chen K., Liu Y., Liu M., Oppenheim J.J., Wang J.M. (2013). Monocyte chemoattractant protein-1/CCL2 produced by stromal cells promotes lung metastasis of 4T1 murine breast cancer cells. PLoS ONE.

[137] Dwyer R.M., Potter-Beirne S.M., Harrington K.A., Lowery A.J., Hennessy E., Murphy J.M., Barry F.P., O’Brien T., Kerin M.J. (2007). Monocyte chemotactic protein-1 secreted by primary breast tumors stimulates migration of mesenchymal stem cells. Clin. Cancer Res..

[138] Valkovic T., Lucin K., Krstulja M., Dobi-Babic R., Jonjic N. (1998). Expression of monocyte chemotactic protein-1 in human invasive ductal breast cancer. Pathol. Res. Pract..

[139] Fujimoto H., Sangai T., Ishii G., Ikehara A., Nagashima T., Miyazaki M., Ochiai A. (2009). Stromal MCP-1 in mammary tumors induces tumor-associated macrophage infiltration and contributes to tumor progression. Int. J. Cancer.

[140] Ueno T., Toi M., Saji H., Muta M., Bando H., Kuroi K., Koike M., Inadera H., Matsushima K. (2000). Significance of macrophage chemoattractant protein-1 in macrophage recruitment, angiogenesis, and survival in human breast cancer. Clin. Cancer Res..

[141] Goede V., Brogelli L., Ziche M., Augustin H.G. (1999). Induction of inflammatory angiogenesis by monocyte chemoattractant protein-1. Int. J. Cancer.

[142] Lanca T., Costa M.F., Goncalves-Sousa N., Rei M., Grosso A.R., Penido C., Silva-Santos B. (2013). Protective role of the inflammatory CCR2/CCL2 chemokine pathway through recruitment of type 1 cytotoxic gammadelta T lymphocytes to tumor beds. J. Immunol..

[143] Li M., Knight D.A., Snyder L.A., Smyth M.J., Stewart T.J. (2013). A role for CCL2 in both tumor progression and immunosurveillance. Oncoimmunology.

[144] Moore B.B., Paine R., Christensen P.J., Moore T.A., Sitterding S., Ngan R., Wilke C.A., Kuziel W.A., Toews G.B. (2001). Protection from pulmonary fibrosis in the absence of CCR2 signaling. J. Immunol..

[145] Smith R.E., Strieter R.M., Zhang K., Phan S.H., Standiford T.J., Lukacs N.W., Kunkel S.L. (1995). A role for C-C chemokines in fibrotic lung disease. J. Leukoc. Biol..

[146] Inoshima I., Kuwano K., Hamada N., Hagimoto N., Yoshimi M., Maeyama T., Takeshita A., Kitamoto S., Egashira K., Hara N. (2004). Anti-monocyte chemoattractant protein-1 gene therapy attenuates pulmonary fibrosis in mice. Am. J. Physiol. Lung Cell Mol. Physiol..

[147] Krenkel O., Puengel T., Govaere O., Abdallah A.T., Mossanen J.C., Kohlhepp M., Liepelt A., Lefebvre E., Luedde T., Hellerbrand C. (2018). Therapeutic inhibition of inflammatory monocyte recruitment reduces steatohepatitis and liver fibrosis. Hepatology.

[148] Gharaee-Kermani M., Denholm E.M., Phan S.H. (1996). Costimulation of fibroblast collagen and transforming growth factor beta1 gene expression by monocyte chemoattractant protein-1 via specific receptors. J. Biol. Chem..

[149] Lyons R.M., Gentry L.E., Purchio A.F., Moses H.L. (1990). Mechanism of activation of latent recombinant transforming growth factor beta 1 by plasmin. J. Cell Biol..

[150] Todorovic V., Jurukovski V., Chen Y., Fontana L., Dabovic B., Rifkin D.B. (2005). Latent TGF-beta binding proteins. Int. J. Biochem. Cell Biol..

[151] Massague J. (2000). How cells read TGF-beta signals. Nat. Rev. Mol. Cell Biol..

[152] Pierce D.F., Gorska A.E., Chytil A., Meise K.S., Page D.L., Coffey R.J., Moses H.L. (1995). Mammary tumor suppression by transforming growth factor beta 1 transgene expression. Proc. Natl. Acad. Sci. USA.

[153] Gorska A.E., Jensen R.A., Shyr Y., Aakre M.E., Bhowmick N.A., Moses H.L. (2003). Transgenic mice expressing a dominant-negative mutant type II transforming growth factor-beta receptor exhibit impaired mammary development and enhanced mammary tumor formation. Am. J. Pathol..

[154] Sun X., Bernhardt S.M., Glynn D.J., Hodson L.J., Woolford L., Evdokiou A., Yan C., Du H., Robertson S.A., Ingman W.V. (2021). Attenuated TGFB signalling in macrophages decreases susceptibility to DMBA-induced mammary cancer in mice. Breast Cancer Res..

[155] Bachman K.E., Park B.H. (2005). Duel nature of TGF-beta signaling: Tumor suppressor vs. tumor promoter. Curr. Opin. Oncol..

[156] Moses H., Barcellos-Hoff M.H. (2011). TGF-beta biology in mammary development and breast cancer. Cold Spring Harb. Perspect. Biol..

[157] Siegel P.M., Massague J. (2003). Cytostatic and apoptotic actions of TGF-beta in homeostasis and cancer. Nat. Rev. Cancer.

[158] Heldin C.H., Landstrom M., Moustakas A. (2009). Mechanism of TGF-beta signaling to growth arrest, apoptosis, and epithelial-mesenchymal transition. Curr. Opin. Cell Biol..

[159] Shi X.P., Miao S., Wu Y., Zhang W., Zhang X.F., Ma H.Z., Xin H.L., Feng J., Wen A.D., Li Y. (2013). Resveratrol sensitizes tamoxifen in antiestrogen-resistant breast cancer cells with epithelial-mesenchymal transition features. Int. J. Mol. Sci..

[160] Wendt M.K., Allington T.M., Schiemann W.P. (2009). Mechanisms of the epithelial-mesenchymal transition by TGF-beta. Future Oncol..

[161] Muraoka-Cook R.S., Kurokawa H., Koh Y., Forbes J.T., Roebuck L.R., Barcellos-Hoff M.H., Moody S.E., Chodosh L.A., Arteaga C.L. (2004). Conditional overexpression of active transforming growth factor beta1 in vivo accelerates metastases of transgenic mammary tumors. Cancer Res..

[162] Parvani J.G., Galliher-Beckley A.J., Schiemann B.J., Schiemann W.P. (2013). Targeted inactivation of beta1 integrin induces beta3 integrin switching, which drives breast cancer metastasis by TGF-beta. Mol. Biol. Cell.

[163] Miyazono K., Ehata S., Koinuma D. (2012). Tumor-promoting functions of transforming growth factor-beta in progression of cancer. Ups. J. Med. Sci..

[164] Li C., Guo B., Bernabeu C., Kumar S. (2001). Angiogenesis in breast cancer: The role of transforming growth factor beta and CD105. Microsc. Res. Tech..

[165] Sime P.J., Xing Z., Graham F.L., Csaky K.G., Gauldie J. (1997). Adenovector-mediated gene transfer of active transforming growth factor-beta1 induces prolonged severe fibrosis in rat lung. J. Clin. Investig..

[166] Sanderson N., Factor V., Nagy P., Kopp J., Kondaiah P., Wakefield L., Roberts A.B., Sporn M.B., Thorgeirsson S.S. (1995). Hepatic expression of mature transforming growth factor beta 1 in transgenic mice results in multiple tissue lesions. Proc. Natl. Acad. Sci. USA.

[167] Fukasawa H., Yamamoto T., Suzuki H., Togawa A., Ohashi N., Fujigaki Y., Uchida C., Aoki M., Hosono M., Kitagawa M. (2004). Treatment with anti-TGF-beta antibody ameliorates chronic progressive nephritis by inhibiting Smad/TGF-beta signaling. Kidney Int..

[168] Nakamura T., Sakata R., Ueno T., Sata M., Ueno H. (2000). Inhibition of transforming growth factor beta prevents progression of liver fibrosis and enhances hepatocyte regeneration in dimethylnitrosamine-treated rats. Hepatology.

[169] Teekakirikul P., Eminaga S., Toka O., Alcalai R., Wang L., Wakimoto H., Nayor M., Konno T., Gorham J.M., Wolf C.M. (2010). Cardiac fibrosis in mice with hypertrophic cardiomyopathy is mediated by non-myocyte proliferation and requires Tgf-beta. J. Clin. Investig..

[170] Denton C.P., Abraham D.J. (2001). Transforming growth factor-beta and connective tissue growth factor: Key cytokines in scleroderma pathogenesis. Curr. Opin. Rheumatol..

[171] Verrecchia F., Mauviel A. (2007). Transforming growth factor-beta and fibrosis. World J. Gastroenterol..

[172] Lee E., Van den Berg D., Hsu C., Ursin G., Koh W.P., Yuan J.M., Stram D.O., Yu M.C., Wu A.H. (2013). Genetic variation in Transforming Growth Factor beta 1 and mammographic density in Singapore Chinese women. Cancer Res..

[173] Khan A.A., Alsahli M.A., Rahmani A.H. (2018). Myeloperoxidase as an Active Disease Biomarker: Recent Biochemical and Pathological Perspectives. Med. Sci..

[174] Van Der Vliet A., Nguyen M.N., Shigenaga M.K., Eiserich J.P., Marelich G.P., Cross C.E. (2000). Myeloperoxidase and protein oxidation in cystic fibrosis. Am. J. Physiol. Lung Cell Mol. Physiol..

[175] Pulli B., Ali M., Iwamoto Y., Zeller M.W., Schob S., Linnoila J.J., Chen J.W. (2015). Myeloperoxidase-Hepatocyte-Stellate Cell Cross Talk Promotes Hepatocyte Injury and Fibrosis in Experimental Nonalcoholic Steatohepatitis. Antioxid. Redox. Signal..

[176] Colon S., Luan H., Liu Y., Meyer C., Gewin L., Bhave G. (2019). Peroxidasin and eosinophil peroxidase, but not myeloperoxidase, contribute to renal fibrosis in the murine unilateral ureteral obstruction model. Am. J. Physiol. Renal. Physiol..

[177] Koller D.Y., Nilsson M., Enander I., Venge P., Eichler I. (1998). Serum eosinophil cationic protein, eosinophil protein X and eosinophil peroxidase in relation to pulmonary function in cystic fibrosis. Clin. Exp. Allergy.

[178] DeNichilo M.O., Panagopoulos V., Rayner T.E., Borowicz R.A., Greenwood J.E., Evdokiou A. (2015). Peroxidase enzymes regulate collagen extracellular matrix biosynthesis. Am. J. Pathol..

[179] Samoszuk M.K., Nguyen V., Gluzman I., Pham J.H. (1996). Occult deposition of eosinophil peroxidase in a subset of human breast carcinomas. Am. J. Pathol..

[180] Hennigan K., Conroy P.J., Walsh M.T., Amin M., O’Kennedy R., Ramasamy P., Gleich G.J., Siddiqui Z., Glynn S., McCabe O. (2016). Eosinophil peroxidase activates cells by HER2 receptor engagement and β1-integrin clustering with downstream MAPK cell signaling. Clin. Immunol..

[181] Walsh M.T., Connell K., Sheahan A.M., Gleich G.J., Costello R.W. (2011). Eosinophil peroxidase signals via epidermal growth factor-2 to induce cell proliferation. Am. J. Respir. Cell Mol. Biol..

[182] Bradley J.R. (2008). TNF-mediated inflammatory disease. J. Pathol..

[183] Locksley R.M., Killeen N., Lenardo M.J. (2001). The TNF and TNF receptor superfamilies: Integrating mammalian biology. Cell.

[184] Bozcuk H., Uslu G., Samur M., Yildiz M., Ozben T., Ozdogan M., Artac M., Altunbas H., Akan I., Savas B. (2004). Tumour necrosis factor-alpha, interleukin-6, and fasting serum insulin correlate with clinical outcome in metastatic breast cancer patients treated with chemotherapy. Cytokine.

[185] Tripsianis G., Papadopoulou E., Anagnostopoulos K., Botaitis S., Katotomichelakis M., Romanidis K., Kontomanolis E., Tentes I., Kortsaris A. (2014). Coexpression of IL-6 and TNF-alpha: Prognostic significance on breast cancer outcome. Neoplasma.

[186] Cai X., Cao C., Li J., Chen F., Zhang S., Liu B., Zhang W., Zhang X., Ye L. (2017). Inflammatory factor TNF-α promotes the growth of breast cancer via the positive feedback loop of TNFR1/NF-κB (and/or p38)/p-STAT3/HBXIP/TNFR1. Oncotarget.

[187] Wolczyk D., Zaremba-Czogalla M., Hryniewicz-Jankowska A., Tabola R., Grabowski K., Sikorski A.F., Augoff K. (2016). TNF-alpha promotes breast cancer cell migration and enhances the concentration of membrane-associated proteases in lipid rafts. Cell Oncol..

[188] Kim S., Choi J.H., Kim J.B., Nam S.J., Yang J.H., Kim J.H., Lee J.E. (2008). Berberine suppresses TNF-alpha-induced MMP-9 and cell invasion through inhibition of AP-1 activity in MDA-MB-231 human breast cancer cells. Molecules.

[189] Pirianov G., Colston K.W. (2001). Interactions of vitamin D analogue CB1093, TNFalpha and ceramide on breast cancer cell apoptosis. Mol. Cell Endocrinol..

[190] Zhang Z., Lin G., Yan Y., Li X., Hu Y., Wang J., Yin B., Wu Y., Li Z., Yang X.P. (2018). Transmembrane TNF-alpha promotes chemoresistance in breast cancer cells. Oncogene.

[191] Wu X., Wu M.-Y., Jiang M., Zhi Q., Bian X., Xu M.-D., Gong F.-R., Hou J., Tao M., Shou L.-M. (2017). TNF-α sensitizes chemotherapy and radiotherapy against breast cancer cells. Cancer Cell Int..

[192] Reeves K.W., Weissfeld J.L., Modugno F., Diergaarde B. (2011). Circulating levels of inflammatory markers and mammographic density among postmenopausal women. Breast Cancer Res. Treat..

[193] Toriola A.T., Dang H.X., Hagemann I.S., Appleton C.M., Colditz G.A., Luo J., Maher C.A. (2017). Increased breast tissue receptor activator of nuclear factor-kappaB ligand (RANKL) gene expression is associated with higher mammographic density in premenopausal women. Oncotarget.

[194] Distler J.H., Schett G., Gay S., Distler O. (2008). The controversial role of tumor necrosis factor alpha in fibrotic diseases. Arthritis Rheum..

[195] Vilcek J., Palombella V.J., Henriksen-DeStefano D., Swenson C., Feinman R., Hirai M., Tsujimoto M. (1986). Fibroblast growth enhancing activity of tumor necrosis factor and its relationship to other polypeptide growth factors. J. Exp. Med..

[196] Theiss A.L., Simmons J.G., Jobin C., Lund P.K. (2005). Tumor necrosis factor (TNF) alpha increases collagen accumulation and proliferation in intestinal myofibroblasts via TNF receptor 2. J. Biol. Chem..

[197] Greenwel P., Tanaka S., Penkov D., Zhang W., Olive M., Moll J., Vinson C., Di Liberto M., Ramirez F. (2000). Tumor necrosis factor alpha inhibits type I collagen synthesis through repressive CCAAT/enhancer-binding proteins. Mol. Cell Biol..

[198] Pierce B.L., Ballard-Barbash R., Bernstein L., Baumgartner R.N., Neuhouser M.L., Wener M.H., Baumgartner K.B., Gilliland F.D., Sorensen B.E., McTiernan A. (2009). Elevated biomarkers of inflammation are associated with reduced survival among breast cancer patients. J. Clin. Oncol..

[199] Cuzick J., Otto F., Baron J.A., Brown P.H., Burn J., Greenwald P., Jankowski J., La Vecchia C., Meyskens F., Senn H.J. (2009). Aspirin and non-steroidal anti-inflammatory drugs for cancer prevention: An international consensus statement. Lancet Oncol..

[200] Harris R.E., Beebe-Donk J., Doss H., Burr Doss D. (2005). Aspirin, ibuprofen, and other non-steroidal anti-inflammatory drugs in cancer prevention: A critical review of non-selective COX-2 blockade (review). Oncol. Rep..

[201] Chew G.L., Huang D., Huo C.W., Blick T., Hill P., Cawson J., Frazer H., Southey M.D., Hopper J.L., Henderson M.A. (2013). Dynamic changes in high and low mammographic density human breast tissues maintained in murine tissue engineering chambers during various murine peripartum states and over time. Breast Cancer Res. Treat..

[202] Ozhand A., Lee E., Wu A.H., Ellingjord-Dale M., Akslen L.A., McKean-Cowdin R., Ursin G. (2013). Variation in inflammatory cytokine/growth-factor genes and mammographic density in premenopausal women aged 50–55. PLoS ONE.

[203] Esbona K., Inman D., Saha S., Jeffery J., Schedin P., Wilke L., Keely P. (2016). COX-2 modulates mammary tumor progression in response to collagen density. Breast Cancer Res..

[204] McTiernan A., Wang C.Y., Sorensen B., Xiao L., Buist D.S., Aiello Bowles E.J., White E., Rossing M.A., Potter J., Urban N. (2009). No effect of aspirin on mammographic density in a randomized controlled clinical trial. Cancer Epidemiol. Biomark. Prev..

[205] Stone J., Willenberg L., Apicella C., Treloar S., Hopper J. (2012). The association between mammographic density measures and aspirin or other NSAID use. Breast Cancer Res. Treat..

[206] Terry M.B., Buist D.S., Trentham-Dietz A., James-Todd T.M., Liao Y. (2008). Nonsteroidal anti-inflammatory drugs and change in mammographic density: A cohort study using pharmacy records on over 29,000 postmenopausal women. Cancer Epidemiol. Biomark. Prev..

[207] Wood M.E., Sprague B.L., Oustimov A., Synnstvedt M.B., Cuke M., Conant E.F., Kontos D. (2017). Aspirin use is associated with lower mammographic density in a large screening cohort. Breast Cancer Res. Treat..

[208] Hopper J.L., Nguyen T.L., Li S. (2021). RE: Chemopreventive Agents to Reduce Mammographic Breast Density in Premenopausal Women: A Systematic Review of Clinical Trials. JNCI Cancer Spectr..

